# Trehalose upregulates progranulin expression in human and mouse models of *GRN* haploinsufficiency: a novel therapeutic lead to treat frontotemporal dementia

**DOI:** 10.1186/s13024-016-0114-3

**Published:** 2016-06-24

**Authors:** Christopher J. Holler, Georgia Taylor, Zachary T. McEachin, Qiudong Deng, William J. Watkins, Kathryn Hudson, Charles A. Easley, William T. Hu, Chadwick M. Hales, Wilfried Rossoll, Gary J. Bassell, Thomas Kukar

**Affiliations:** Department of Pharmacology, Emory University, School of Medicine, 1510 Clifton Rd, Atlanta, GA 30322 USA; Laboratory of Translational Cell Biology, Emory University, School of Medicine, Atlanta, GA 30322 USA; Department of Cell Biology, Emory University, School of Medicine, Atlanta, GA 30322 USA; Coulter Department of Biomedical Engineering, Georgia Institute of Technology, 313 Ferst Drive, Atlanta, GA 30332 USA; Center for Neurodegenerative Disease, Emory University, School of Medicine, Atlanta, GA 30322 USA; Department of Neurology, Emory University, School of Medicine, Atlanta, GA 30322 USA

**Keywords:** Frontotemporal lobar degeneration, Frontotemporal dementia, Alzheimer’s disease, Parkinson’s disease, Neurodegeneration, Progranulin, Trehalose, Autophagy, Ubiquitin, TDP-43, Lysosome, Lysosome storage disease, Neuronal ceroid lipofuscinosis, TFEB

## Abstract

**Background:**

Progranulin (PGRN) is a secreted growth factor important for neuronal survival and may do so, in part, by regulating lysosome homeostasis. Mutations in the PGRN gene (*GRN*) are a common cause of frontotemporal lobar degeneration (FTLD) and lead to disease through PGRN haploinsufficiency. Additionally, complete loss of PGRN in humans leads to neuronal ceroid lipofuscinosis (NCL), a lysosomal storage disease. Importantly, *Grn−/−* mouse models recapitulate pathogenic lysosomal features of NCL. Further, *GRN* variants that decrease PGRN expression increase the risk of developing Alzheimer’s disease (AD) and Parkinson’s disease (PD). Together these findings demonstrate that insufficient PGRN predisposes neurons to degeneration. Therefore, compounds that increase PGRN levels are potential therapeutics for multiple neurodegenerative diseases.

**Results:**

Here, we performed a cell-based screen of a library of known autophagy-lysosome modulators and identified multiple novel activators of a human *GRN* promoter reporter including several common mTOR inhibitors and an mTOR-independent activator of autophagy, trehalose. Secondary cellular screens identified trehalose, a natural disaccharide, as the most promising lead compound because it increased endogenous PGRN in all cell lines tested and has multiple reported neuroprotective properties. Trehalose dose-dependently increased *GRN* mRNA as well as intracellular and secreted PGRN in both mouse and human cell lines and this effect was independent of the transcription factor EB (TFEB). Moreover, trehalose rescued PGRN deficiency in human fibroblasts and neurons derived from induced pluripotent stem cells (iPSCs) generated from *GRN* mutation carriers. Finally, oral administration of trehalose to *Grn* haploinsufficient mice significantly increased PGRN expression in the brain.

**Conclusions:**

This work reports several novel autophagy-lysosome modulators that enhance PGRN expression and identifies trehalose as a promising therapeutic for raising PGRN levels to treat multiple neurodegenerative diseases.

**Electronic supplementary material:**

The online version of this article (doi:10.1186/s13024-016-0114-3) contains supplementary material, which is available to authorized users.

## Background

Progranulin is a multi-functional, secreted glycoprotein involved in cell survival, modulation of inflammation, and neuroprotection [1, 2]. Mutations in the human *GRN* gene are one of the most common causes of frontotemporal lobar degeneration (FTLD) and the vast majority cause loss of function by decreasing *GRN* mRNA and PGRN protein by at least 50 % via haploinsufficiency [3–5]. Decreased PGRN expression is also implicated as a risk factor for Alzheimer’s disease (AD) and Parkinson’s disease (PD) [6–8]. In the brain, PGRN is expressed predominantly in neurons and microglia. FTLD-GRN pathology is characterized by neurodegeneration, neuroinflammation, and intra-neuronal and glial inclusions containing the TAR DNA-binding protein 43 (TDP-43), the autophagy adaptor protein p62/SQSTM1, and ubiquitinated proteins (reviewed in [9]). Accumulation of these proteins suggests that defects in protein removal systems, such as the autophagy-lysosome pathway, may contribute to disease. In support of this, abnormal accumulation of lysosomal proteins and lipofuscin, an age-related lipid-containing residue of lysosomal digestion, occur in *Grn−/−* mice and human FTLD-GRN brains [10]. Moreover, complete loss of PGRN causes neuronal ceroid lipofuscinosis (NCL) [11], an early-onset lysosomal storage disease. Together, these data indicate that PGRN plays a critical, yet undefined role in lysosome function and homeostasis.

The identification of small molecules to raise PGRN protein levels is an attractive therapeutic strategy for neurodegeneration caused by PGRN deficiency. Currently, there are no clinically approved methods to increase PGRN in patients with FTLD-GRN. In this study, we screened a library of small molecule modulators of the autophagy-lysosome pathway to identify novel enhancers of PGRN expression. The top compounds identified in the screen were further tested in secondary cellular screens and relevant models of *GRN* deficiency, including patient derived cells and an in vivo mouse model for the ability to raise PGRN.

## Methods

### Chemical reagents

Bafilomycin A1 (BafA1), PP242, and Torin1 were obtained from Tocris (R&D Systems, Minneapolis, MN). Chloroquine diphosphate and Actinomycin D (ActD) were obtained from Sigma (St. Louis, MO). Suberanilohydroxamic acid (SAHA or vorinostat) and rapamycin were obtained from LC Laboratories (Woburn, MA). Trehalose (dihydrate) was obtained from Sigma or Brooklyn Premium (Brooklyn, NY). BafA1, PP242, Torin1, rapamycin, and SAHA were dissolved in DMSO and stocks were frozen at −20 °C. Chloroquine and trehalose were dissolved in ultrapure Milli-Q water (EMD Millipore) and frozen at −20 °C or filtered (0.22-μm) and stored at 4 °C, respectively. Trehalose stocks were made up fresh as needed.

### Cell culture

Human embryonic kidney cells (HEK293T; American Type Culture Collection), human HeLa cells (American Type Culture Collection), human neuroglioma cells (H4; American Type Culture Collection), and mouse neuroblastoma cells (N2a; American Type Culture Collection) were cultured in high glucose Dulbecco’s Modified Eagle’s Medium (DMEM) supplemented with 10 % FBS, 1 % penicillin/streptomycin, and 1 % Gluta-max. Human neuroblastoma cells (SH-SY5Y; American Type Culture Collection) were cultured in DMEM/Ham’s F12 1:1 medium supplemented with 10 % FBS and 1 % penicillin/streptomycin. The TFEB-GFP stable HeLa cell line [12] was a kind gift provided by Dr. Shawn Ferguson (Yale University) and was cultured in high glucose Dulbecco’s Modified Eagle’s Medium (DMEM) supplemented with 10 % FBS, 1 % penicillin/streptomycin, and 1 % Gluta-max. The HAP1 human haploid cell lines were purchased from Horizon Discovery Group. The TFEB knock-out cell line was produced by CRISPR/Cas9 gene editing which introduced a frameshift mutation into the coding sequence (7 bp deletion in exon 2) of TFEB. HAP1 cells were cultured in Iscove’s Modified Dulbecco’s Medium (IMDM) supplemented with 10 % FBS and 1 % penicillin/streptomycin. Primary mouse cortical neurons (embryonic day 18) were a generous gift from Dr. Randy Hall (Emory University). All cell lines were maintained at 37 °C with 5 % CO_2_.

### Human fibroblast biopsy and culture

Human dermal fibroblasts were collected under protocol 00064365 as approved by the Emory University Institutional Review Board. Informed written consent was obtained for all research subjects. A 2-mm dermal punch biopsy was taken from each subject under sterile conditions. The biopsy sample was placed in culture medium (DMEM with 4.5 mg/mL glucose, L-glutamine, and sodium pyruvate) with or without 0.6 % v/v fungizone (Gibco). Culture media was supplemented with 10 % FBS (Atlanta Biologicals) and 1 % penicillin/streptomycin (Gibco) and filtered through a 0.2-μm syringe filter prior to use. In a culture hood, skin biopsies were rinsed 3× with sterile PBS and the skin biopsy was then transferred to a single well of a 6-well culture dish in ~50-100 μL PBS or culture medium. The tissue was cut into several smaller pieces using a sterile rounded-tip scalpel blade. A sterile glass coverslip (25 mm) was placed on top of the skin pieces and pressed down onto the dish to secure the sample. Carefully, 3 mL of culture medium was added to the well and the plate was placed in an incubator at 37 °C with 5 % CO2. The cultures were allowed to sit undisturbed for 6–7 days and then half of the media was removed and replaced with fresh media. Fibroblasts typically emerged by day 10 and were ready to passage by day 12–16. Fibroblast cultures were passaged as needed using 0.25 % trypsin with EDTA (Invitrogen) and gradually scaled up to larger culture dishes in culture medium without fungizone. Individual cell plugs from each line were then cryopreserved at low passage numbers, typically at *P* < 5. Fibroblast lines were generated from 3 patients with the following *GRN* mutations: c.1477C > T (R493X) (designated GRN #1, GRN #2) and c.592_593delAG (R198GfsX19) (designated GRN #3).

### Induced pluripotent stem cell (iPSC) generation and characterization

One control and one GRN (GRN #3) fibroblast line were reprogrammed into iPSCs using the Cytotune 2.0 Kit (Life Technologies) per the manufacturer’s protocol. In brief, early passage fibroblast (*P* < 10) were grown to approximately 50–80 % confluency in fibroblast medium consisting of 10 % ES-qualified FBS (Life Technologies, 0.1 mM NEAA, 55 μM β-mercaptoethanol, high glucose DMEM (Life Technologies). On Day 0, fibroblasts were transduced with three Sendai viruses encoding Klf4–Oct3/4–Sox2 (KOS), hc-Myc, and hKlf4, each at an MOI of 5). Cells were fed with fibroblast medium every other day for 7 days. On day 7, cells were passaged onto vitronectin (Life Technologies) coated dishes at a density of 2.5 × 10^5^ - 5.0 × 10^5^ cells/well. Beginning on day 8, cells were fed every day in Essential 8 medium (Life Technologies). Resulting iPS colonies were manually picked and transferred to a dish coated with either vitronectin or Matrigel (BD). iPSCs were maintained on Matrigel coated dishes and mTesR1 medium (Stem Cell Technologies) and passaged every 5–7 days.

### iPSC neuronal differentiation

Prior to differentiation, iPSC colonies were treated with 10 μM ROCK inhibitor, Y-27632 (Stem Cell Technologies), for ~1 h. iPSCs were then treated with Accutase (Stem Cell Technologies) for ~8 min to obtain a single cell suspension. Cells were spun out of Accutase and resuspended in N2B27 differentiation medium (1:1 Advanced DMEM-F12/Neurobasal, 1× N2, 1× B27, 0.2 % Penstrep, 1× Glutamax, 110 μM β-mercaptoethanol) and seeded in 10 cm Ultra-Low Attachment dishes (Corning) in order to form embryoid bodies. Cells were maintained as embryoid bodies throughout the differentiation and were fed every 2 days. For the first 2 days, the differentiation medium contained a final concentration of 3 μM CHIR99021 (Stem Cell Technologies), 10 μM SB431542 (Stem Cell Technologies), 10 μM LDN193189 (Stemgent), 0.4 μg/mL Ascorbic Acid (Sigma), 10 μM Y-27632. On Day 2, 1 μM Retinoic Acid (Sigma) and 500 nM Smoothened Agonist (Millipore) were added to the medium. On Day 4, CHIR99021 was removed from the medium. On Day 8, SB and LDN were removed from the medium and the following supplements were added: 10 ng/mL BDNF (Peprotech), 10 ng/mL GDNF (Peprotech), and 10 μM DAPT (Tocris). On day 18, embryoid bodies were disassociated to single cells using papain/DNase (Worhtington Bio) and plated on polyornithine/laminin coated glass coverslips or cell culture plates. Neurons were typically used for experiments around 1-week post-plating.

### Chemical library screen

HEK293T cells were plated in a 96-well plate at approximately 1.5 × 10^4^ cells per well in complete medium. The following day, cells were transfected with 100 ng of the GLuc-ON human PGRN promoter (Gene Accession: NM_001012479; promoter length: 1253 bp; sequence verified) dual reporter (GLuc/SEAP) plasmid (Genecopoeia) using Mirrus LT1 transfection reagent. 24 h after transfection, a commercially available library of ~100 reported chemical enhancers or inhibitors of the autophagy pathway (Enzo Screen-Well library; BML-2837) were applied to the cells overnight for 16 h. Final concentration for all drugs were 1 μM except for bafilomycin A1 (50 nM), chloroquine (50 μM), and trehalose (100 mM) based on previously published reports for increasing progranulin expression or activating autophagy (see main text). After treatment, the media was collected and transferred to a new 96-well plate, spun briefly to remove cell debris, and 10 μL of each sample was transferred to a new 96-well black plate. Secreted GLuc activity normalized to SEAP was measured for each compound using the SecretePair Dual Luminescence Assay Kit (GeneCopoeia) on a BioTek Synergy plate reader and compared to mock-treated (DMSO) cells. The screen was performed in two independent experiments and the average fold-increase compared to mock treatment is reported. Staurosporine (1 μM) was used as a control for overt cell toxicity and compounds whose individual GLuc or seAP values were below those of staurosporine were omitted.

### Cell lysis and Western blotting

Cells were rinsed 2× in PBS and lysed in ice-cold RIPA buffer (50 mM Tris–HCl, pH 8.0, 150 mM NaCl, 1 % Triton-×100, 0.1 % SDS, 0.5 % sodium deoxycholate) in the presence of protease and phosphatase inhibitor cocktail (PPIC, Pierce). RIPA lysates were sonicated at 20 % amplitude for 5 cycles of 2 s on/2 s off on ice using a sonic dismembrator (QSonica, LLC; Newton, Ct). In some experiments cells were first lysed in CYTO buffer (50 mM Tris–HCl, pH 8.0, 150 mM NaCl, 0.5 % Triton X-100 + PPIC) on ice for 10 min followed by centrifugation at max speed for 10 min at 4 °C. The resultant supernatant contained cytoplasmic and membrane proteins. The pellet was rinsed with CYTO buffer, re-centrifuged for 2 min and the supernatant was discarded. The pellet was then resuspended in NUC buffer (RIPA; 50 mM Tris–HCl, pH 8.0, 150 mM NaCl, 1 % Triton-×100, 0.1 % SDS, 0.5 % sodium deoxycholate + PPIC) and sonicated as above to obtain a nuclear extract. GAPDH and Histone H3 were used as protein markers for cytoplasmic/membrane and nuclear extraction efficiencies, respectively. Total protein was measured by BCA assay (Pierce) and Western blot samples were prepared with 4× loading buffer (125 mM Tris, pH 6.8, 8 % LDS, 40 % glycerol, Orange G) and heat-denatured at 70 °C for 15 min. Samples of equal protein were run on a range of Bio-Rad TGX mini-gels and transferred to PVDF or Nitrocellulose membranes. Membranes were blocked with LiCor Odyssey blocking buffer (1:1 TBS/Blocking Buffer) for 1 h at room temperature followed by incubation with primary antibody (diluted in 1:1 TBST/Blocking Buffer) over night at 4 °C with gentle rocking. HRP-conjugated secondaries (Jackson Labs or Cell Signaling technologies) diluted in 5 % milk/TBST or LiCor fluorescent secondaries diluted in 1:1 TBST/Blocking Buffer were used. West Dura (Pierce) substrate was used for chemiluminescent detection. Blots were imaged using an Odyssey Fc (LiCor) and analyzed using Image Studio software (Ver 3.1) for densitometry analysis. The following primary antibodies were used for Western blot: LC3A/B (1:1000; CST), total 4EBP1 and P-4EBP1 (1:1000; CST), human PGRN (1:1000; R&D), PGRN/PCDGF (1:700; Invitrogen), mouse PGRN (1 μg/mL; R&D), total S6 and P-S6 (1:1000; CST), p62 (1:1000; BD), TFEB (1:1000; CST), TUJ1 (1:2,000; Covance). Tubulin (1:20,000; Epitomics), Actin (1:10,000; Epitomics), GAPDH (1:5,000; Sigma), and Histone H3 (1:5,000; Millipore) were used as loading controls.

### Secreted progranulin measurement

Cells were plated in 6-cm dishes in complete medium. Two days after plating, cells were washed 2× with serum-free media and treated over night with vehicle or drug at the indicated concentrations in serum-free media. Cell supernatants were collected the following day at the indicated times and immediately spun at 6000 rpm for 5 min at 4 °C to clear debris. The supernatant (500 μL) was transferred to a 0.5 mL Amicon Ultra concentrator (50 kDa cutoff) and spun for 5–10 min at 14,000 × g. The spin filter was placed upside down in a fresh tube and spun at 1000 × g for 2 min to collect the concentrated sample. The concentrated samples were normalized to total protein in the cell lysates to account for differences in cell numbers, mixed with 4× loading buffer to a final concentration of 1×, and heated at 70 °C for 15 min to denature.

### Immunocytochemistry

H4 or primary fibroblast cells were fixed with 4 % paraformaldehyde for 15 min. Cells were then permeabilized with ice-cold methanol for 10 min. After blocking with 0.1 % BSA in PBS, cells were incubated with goat anti-PGRN (1:300; R&D) in blocking buffer overnight at 4 °C. After washing with PBS, cells were incubated in secondary antibodies conjugated to Cy3 (1:300; Jackson Immunoresearch) and DAPI (1:1000; Life Technologies) for 1 h at room temperature. Slides were mounted using Vectashield Hard Set (Vector Laboratories, Inc.; Burlingame, CA). iPSC-neurons were fixed with 4 % paraformaldehyde for 15 min. Cells were then permeabilized with ice-cold methanol or 0.1 % triton X-100 in PBS for 10 min. After blocking with 0.1 % BSA or 5 % Normal Donkey Serum (NDS), cells were incubated with the following primary antibodies in blocking buffer overnight at 4 °C: PGRN (Goat polyclonal, 1:300; R&D), LC3A/B (1:100; CST), Tuj1 (1:2000; Covance). After washing with PBS, cells were incubated in secondary antibodies conjugated to Cy5 (1:300; Jackson Immunoresearch) or Alexa fluor 488 (1:300; Life Technologies) for 1 h at room temperature. Slides were mounted using ProLong Gold Antifade Reagent with DAPI (Life Technologies). Images were collected with a Zeiss LSM 510 NLO META system (Emory University Integrated Cellular imaging Microscopy Core) or EVOS FL Cell Imaging System (Life Technologies).

### Quantitative real-time PCR

RNA was extracted using QiaShredder lysis buffer and purified using RNeasy spin columns from Qiagen. Purified RNA (900 ng) was used as a template to produce cDNA using AB high Capacity RNA-to-cDNA kit (Life Technologies) according to the manufacturer’s protocol on a Bio-Rad C1000 Thermo Cycler. Approximately 20 ng of cDNA was used for quantitative real-time PCR (qPCR) experiments using Eppendorf plates and AB Power-SYBR mix (20 μL final reaction volume) on an Eppendorf Mastercycler ep realplex S. Primers were obtained from IDT and used at a final concentration of 200 nM. The following cycle conditions were used for all genes: 95 °C for 10 min followed by 40 cycles of 95 °C for 15 s and 54 °C for 60 s with a final extension step of 95 °C for 15 s and 54 °C for 60 s. The ΔΔCt method was used to calculate fold changes in RNA levels compared to vehicle treated cells after normalization to a reference gene, U36-B. The primer sets used were reported previously [13] as follows: human U36B-F, 5′-CGAGGGCACCTGGAAAAC-3′; human U36B-R, 5′-CACATTCCCCCGGATATGA-3′; human GRN-F, 5′-CAGGGACTTCCAGTTGCTGC-3′; human GRN-R, 5′-GCAGCAGTGATGGCCATCC-3′.

### Enzyme-linked immunosorbent assay (ELISA)

Mouse progranulin ELISAs were purchased from Adipogen (San Diego, CA) and carried out according to the manufacturer’s protocol. Plasma samples were diluted 1:500 in 1× Diluent buffer. Brain lysates were diluted 1:50 in 1× Diluent buffer. All ELISA measurements were done in duplicate and fell within the standard curve generated by the provided recombinant progranulin standard. Mouse plasma and brain lysate samples were randomized and loaded blind to the researcher. For brain and plasma ELISAs, a positive (*Grn*+/+) and negative (*Grn*−/−) control sample were also run to verify specificity of the ELISA.

### Animal subjects and experiments

All animal work was conducted with prior Institutional Animal Care and Use Committee (IACUC) approval, and was performed in accordance with PHS guidelines. All procedures were performed under conditions designed to minimize pain and distress. Emory University is an Association for Assessment and Accreditation of Laboratory Animal Care (AAALAC) approved institution, and follows the current version of the Guide for the Care and Use of Laboratory Animals (8th Edition), as adopted by the Office of Laboratory Animal Welfare (OLAW). Twenty *Grn* heterozygous mice (*Grn+/−*) obtained from our colony by crossing *Grn* knockout mice (*Grn−/−*) [14] with wild-type C57bl6J (originally acquired from The Jackson Laboratory, Bar Harbour, Maine) were used in the study. We elected to use both male and female mice in approximately equal numbers based on the number of animals available in the colony and to comply with recent NIH guidance on maintaining gender balance in biomedical animal studies. For treatments, solutions of 2 % sucrose or 2 % trehalose were made up in water (same regularly provided to animals in the vivarium) and filtered through a 0.2-μm stericup filter. Fresh water was changed out once per week. Weights of the water bottles were measured before and after each exchange to estimate amount of water consumed per group. Mice were weighed periodically to monitor changes in weight gain per group. After 65 days of treatment, mice were euthanized by decapitation after anesthetization with isoflurane. Blood was collected at time of death in a reservoir containing 100 μL of 0.5 M EDTA. Plasma was separated by centrifugation at 3000 rpm for 15 min at 4 °C and stored at −80 °C. Whole brain was harvested and separated into hemispheres after removal of the cerebellum. One half of the brain was drop fixed in 4 % paraformaldehyde (PFA) and the other half was placed in a tube, snap frozen in liquid nitrogen, and stored at −80 °C. Frozen brain tissue was ground under liquid nitrogen with a mortar and pestle to create a uniform powder. For protein extraction, approximately 50 mg of tissue powder was homogenized in lysis buffer containing 50 mM Tris–HCl (pH 7.4), 150 mM NaCl, 1 % Triton X-100 and complete protease and phosphatase inhibitor cocktail (Pierce) and sonicated briefly. Protein concentrations were determined by BCA assay (Pierce) and Western blot samples were made up as described above. Approximately 25 μg of total protein per sample was loaded onto a 12 % TGX mini gel (Bio-Rad) for Western blotting as described above.

### Statistical analysis

All values are expressed as the mean ± SEM. For experiments where two groups were compared, a standard two-tailed Student’s *t*-test was used to measure significance. For comparisons of more than two groups, one-way analysis of variance (ANOVA) was used followed by Dunnet’s or Tukey’s comparison *post-hoc* test. For correlation analysis, Pearson’s *r* was used. All statistical analyses were performed in GraphPad Prism 6.02 (GraphPad Software, La Jolla, California, USA). A *P*-value <0.05 was considered significant.

## Results

### Identification of novel autophagy-lysosome modulators that increase PGRN

**Fig. 1:**
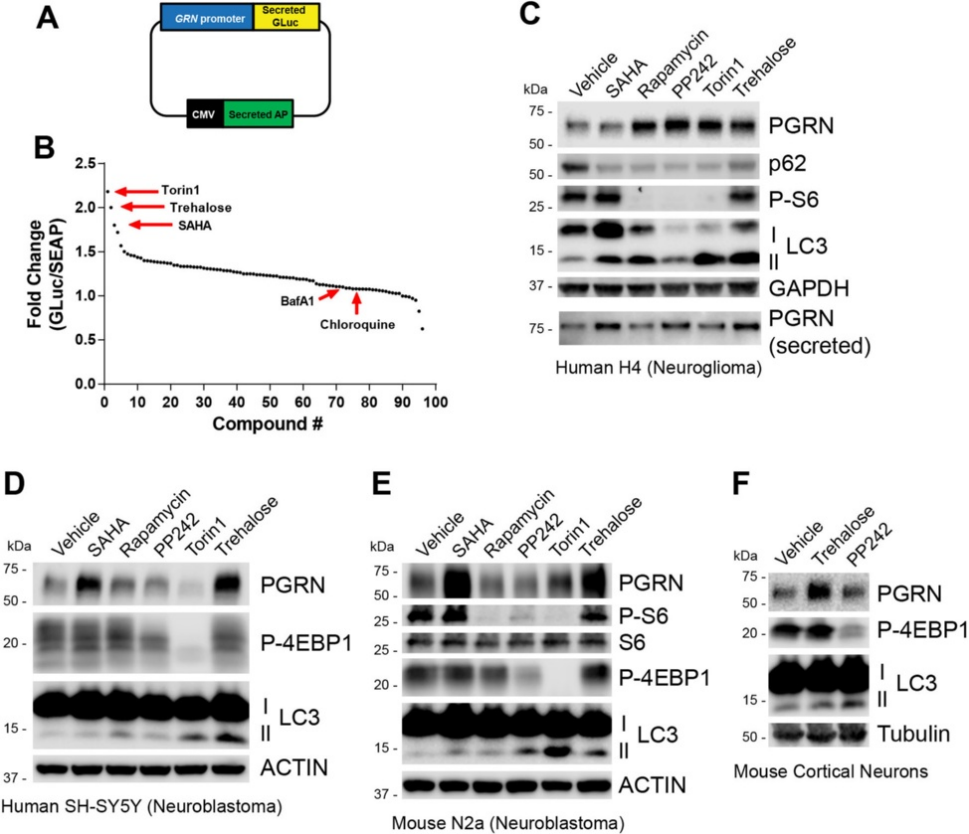
Identification and validation of small molecule autophagy-lysosome modulators that increase PGRN. **a** Schematic of the human *GRN* promoter dual reporter plasmid used for identifying compounds that affect PGRN expression at the transcriptional level. Secreted *Gaussia* Luciferase (GLuc) activity monitors *GRN* promoter activity while secreted Alkaline Phosphatase (SEAP) activity monitors transfection efficiency and is used for normalization. **b** Fold-change in reporter expression activity (GLuc/SEAP) after transfection into HEK293T cells followed by treatment with a custom library of autophagy-lysosome modulators. SAHA (1 μM), which increases *GRN* transcription, was used as a positive control for the reporter assay. Bafilomycin A1 (50 nM) and chloroquine (50 μM), which increase PGRN expression primarily via a post-transcriptional mechanism, were included as negative controls. Trehalose was used at 100 mM, as reported in the literature for inducing autophagy. All other compounds were used at 1 μM. The data presented are the average of two independent replicates of the drug screen. **c** Immunoblot of cell lysates and conditioned media (bottom) from human neuroglioma (H4) cells treated with vehicle, SAHA (1 μM), rapamycin (1 μM), PP242 (1 μM), Torin1 (1 μM), or trehalose (100 mM) for 24 h. Immunoblot of cell lysates from **d** human neuroblastoma cells (SH-SY5Y) cells and **e** mouse neuroblastoma cells (N2a) treated as in c. **f** Immunoblot of cell lysates from primary mouse cortical neurons (E18) treated for 24 h with vehicle, trehalose (100 mM), or PP242 (1 μM). In c-f, p62 and/or LC3-II were used to monitor autophagy induction and P-S6 and/or P-4EBP1 were used to assess mTOR inhibition. Immunoblot images are representative of at least two independent experiments

PGRN partially localizes to late endosomal and lysosomal compartments within neurons [15, 16] and loss of PGRN causes lysosome dysfunction; therefore, we hypothesized that modulators of this pathway may also regulate *GRN* expression [11, 17, 18]. To test this hypothesis, we expressed a dual reporter construct (Fig. 1a) in HEK293T cells to monitor activity of the human *GRN* promoter and screened a custom chemical library of autophagy-lysosome modulators (see Methods section). We identified several compounds that increased reporter activity ~2-fold (Fig. 1b), comparable to suberoylanilide hydroxamic acid (SAHA), a histone deacetylase inhibitor (HDAC) which was previously identified as a robust regulator of *GRN* transcription in a high-throughput screen (HTS) of the Prestwick chemical library [13]. The top hits from our screen were Torin1, a mechanistic target of rapamycin (mTOR) inhibitor [19], and trehalose, an mTOR-independent activator of autophagy [20]. The mTOR kinase is a principle negative regulator of autophagy and numerous autophagy activators have been discovered that work via inhibiting mTOR and downstream signaling pathways (reviewed in [21]). We found that several other compounds that reportedly inhibit mTOR also increased *GRN* promoter activity (Additional file 1: Table S1), suggesting this signaling pathway may be involved in regulating *GRN* expression.

Next, we used a secondary cellular screen to determine whether identified compounds increased endogenous levels of PGRN. We treated human H4 neuroglioma cells with SAHA, trehalose, or the mTOR inhibitors rapamycin, PP242, or Torin1 for 24 h and monitored autophagy activation and PGRN expression by immunoblot (Fig. 1c). The conversion of the microtubule-associated protein light chain 3 from a non-lipidated form (LC3-I) to a lipidated membrane-bound form (LC3-II) correlates with the formation of autophagosomes and is used as a marker for autophagy [22]. All compounds resulted in increased levels of LC3-II and decreased levels of p62, an autophagic substrate, indicating autophagy activation. Trehalose had no effect on phosphorylation of S6 ribosomal protein (P-S6), a downstream target of mTOR, confirming that activation of autophagy was independent of mTOR inhibition. Further, trehalose and mTOR inhibitors increased both intracellular and secreted PGRN (Fig. 1c and Additional file 1: Figure S1a). Importantly, trehalose also increased PGRN in human (Fig. 1d) and mouse (Fig. 1e) neuroblastoma cell lines, and in mouse primary cortical neurons (Fig. 1f). In contrast, mTOR inhibitors failed to increase PGRN in these cell lines even though increased LC3-II was observed (Fig. 1d–f). These data indicate a mechanistic difference between trehalose and mTOR inhibitors in cells of the neuronal lineage and that mTOR-inhibition and PGRN upregulation are independent pathways. Thus, we focused on trehalose as our lead molecule because it had consistent and robust effects on PGRN levels across multiple disease-relevant cell types.

### Trehalose increases PGRN expression via a transcriptional mechanism

Next, we characterized the activity of trehalose in cultured cells in more detail. Using bafilomycin A1 (BafA1) to block autophagosome fusion with lysosomes, we found that trehalose increased autophagic flux in H4 cells (Additional file 1: Figure S2a-b), consistent with previous reports that trehalose likely acts at the step of autophagy initiation [23, 24]. In addition, treatment of H4 cells with trehalose had no overt effect on lysosomal acidification based on LysoTracker Red staining, unlike the vacuolar-ATPase inhibitor BafA1 (Additional file 1: Figure S2c). Therefore, it is likely that trehalose and the previously reported BafA1 have different mechanisms of action for increasing PGRN [25]. Finally, dose- or time-dependent treatment of H4 cells with trehalose did not affect cell viability (Additional file 1: Figure S2d). Overall, these results confirm that trehalose is well tolerated by mammalian cells.

**Fig. 2:**
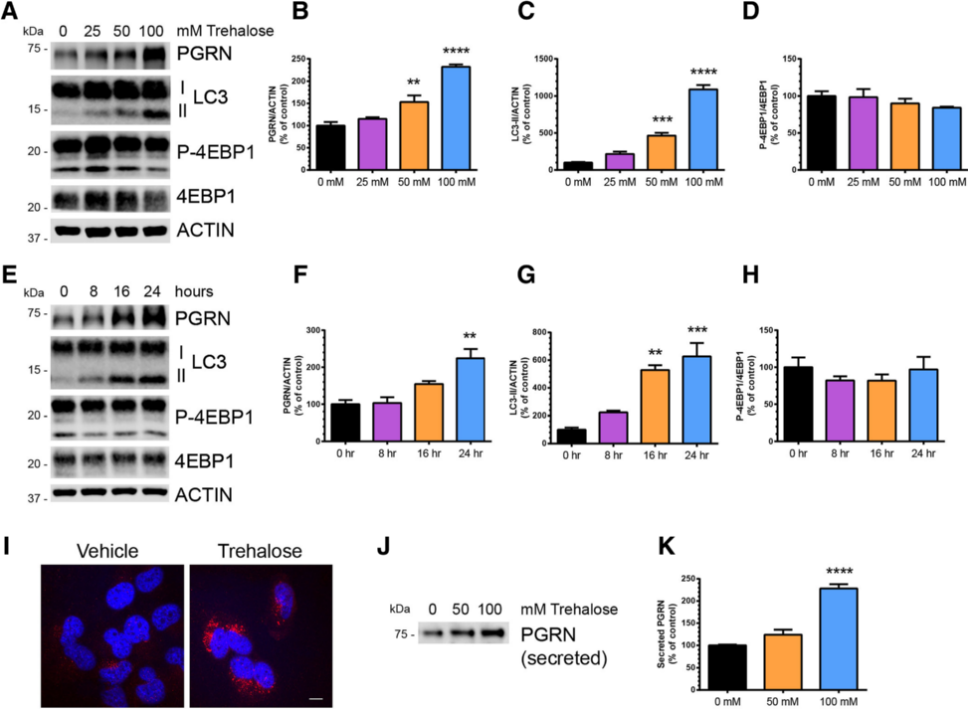
Trehalose induces a dose- and time-dependent increase in PGRN expression in cultured cells. **a** Immunoblot of cell lysates from H4 cells treated with 0, 25, 50, or 100 mM trehalose for 20 h. Quantification of **b** PGRN, **c** LC3-II, and **d** p-4EBP1 immunoreactivty from a (*n* = 3 independent experiments). **e** Immunoblot of cell lysates from H4 cells treated with 100 mM trehalose for 0, 8, 16, or 24 h. Quantification of **f** PGRN, **g** LC3-II, and **h** p-4EBP1 immunoreactivity from e (*n* = 3 independent experiments). **i** Representative image of H4 cells treated with vehicle or trehalose (100 mM) for 24 h followed by staining with an antibody against human PGRN (*red*). Nuclei (*blue*) are stained with DAPI, 4’,6-diamidino-2-phenylindole. Scale bar, 10 μm. **j** Immunoblot of conditioned media from H4 cells treated with 0, 50, or 100 mM trehalose for 20 h. **k** Quantification of secreted PGRN immunoreactivity from Western blots of conditioned media in j (*n* = 3 independent experiments). In all graphs, the bars represent the mean ± SEM. *Differs from control, *P* < 0.05, **P < 0.01, ***P < 0.001, ****P < 0.0001 using one-way ANOVA followed by Dunnett’s comparison *post-hoc* test

**Fig. 3:**
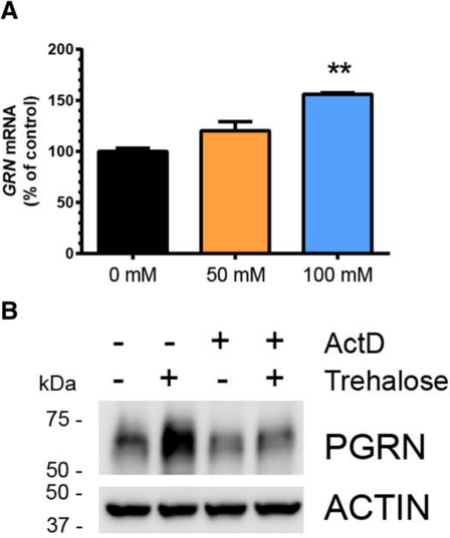
Trehalose increases PGRN expression via increased transcription. **a** *GRN* mRNA levels were quantified from H4 cells after treatment with 0, 50, or 100 mM trehalose for 18 h (*n* = 2 independent experiments run in duplicates). The bars represent the mean ± SEM. **Differs from control, *P* < 0.01 using one-way ANOVA followed by Dunnett’s comparison *post-hoc* test. **b** Immunoblot of cell lysates from H4 cells treated with trehalose (100 mM) plus actinomycin D (1 μM) overnight (16 h) to block new transcription. Results are representative of two independent experiments

Next, we found that trehalose significantly increased PGRN in a dose- (Fig. 2a–b) and time- (Fig. 2e–f) dependent manner that coincided with increased LC3-II (Fig. 2a, c and Fig. 2e, g). Once again, this effect was independent of mTOR inhibition as measured by phosphorylated 4EBP-1, a direct target of mTOR (Fig. 2a, d and Fig. 2e, h). A significant increase in LC3-II was observed prior to an increase in PGRN, indicating that activation of autophagy may precede PGRN upregulation. The dose- and time-dependent effects of trehalose on PGRN were confirmed in both human and mouse neuroblastoma cell lines as well (Additional file 1: Figure S3a-c). H4 cells treated with trehalose showed a robust increase in intracellular PGRN by immunocytochemistry, confirming the immunoblot data (Fig. 2i). Importantly, we observed a dose-dependent increase of secreted PGRN in the media of H4 cells treated with trehalose (Fig. 2j–k). Finally, we found that trehalose increased *GRN* mRNA in a dose-dependent manner in H4 cells (Fig. 3a) and human neuroblastoma cells (Additional file 1: Figure S3d) using quantitative real-time PCR (qPCR). Treatment of H4 cells with actinomycin D, an inhibitor of mRNA transcription, blocked the trehalose-mediated increase of PGRN (Fig. 3b). These data provide further evidence that trehalose primarily acts by increasing *GRN* gene transcription and supports the validity of our initial *GRN* promoter reporter assay.

### The transcription factor EB (TFEB) does not mediate trehalose-induced upregulation of PGRN

**Fig. 4:**
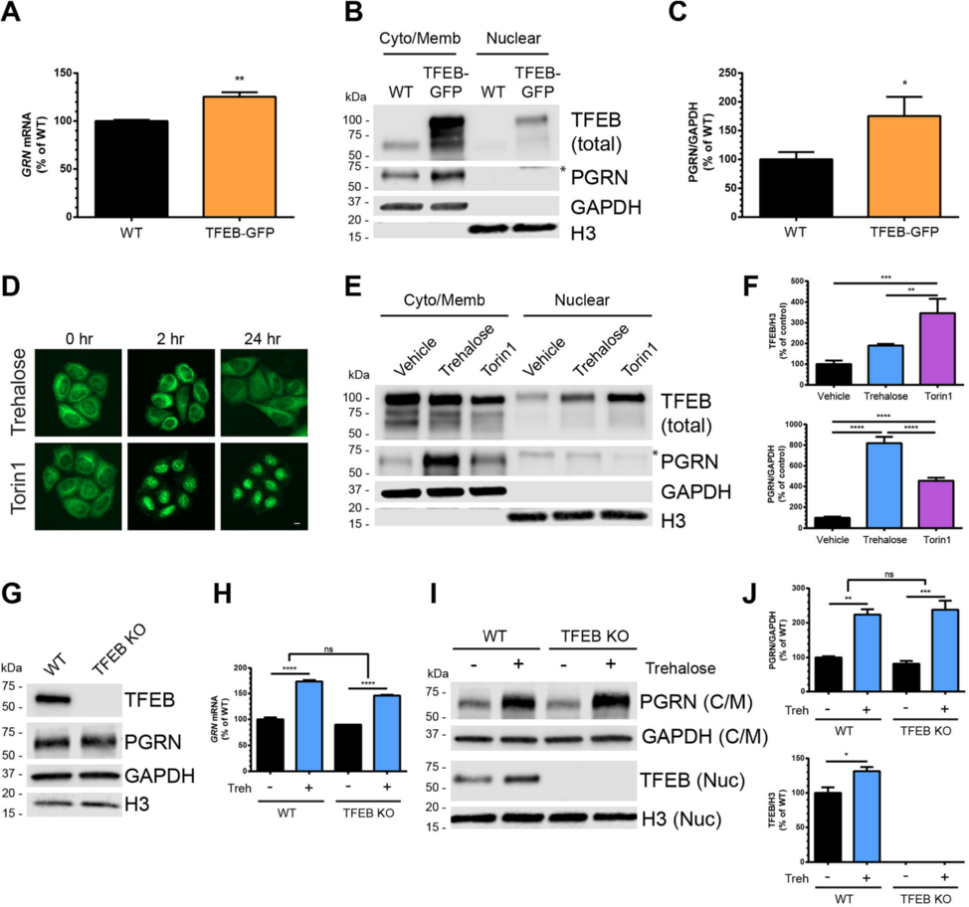
The transcription factor EB (TFEB) does not mediate trehalose-induced upregulation of PGRN. **a** *GRN* mRNA levels were quantified from wild-type HeLa (WT) cells or HeLa cells stably over-expressing TFEB-GFP (TFEB-GFP) (*n* = 3 independent experiments run in duplicate). **b** WT or TFEB-GFP HeLa cells were fractionated into cytoplasmic/membrane and nuclear extracts and immunoblotted for total TFEB and PGRN. GAPDH and H3 were used to verify fractionation efficiency and were used as loading controls. Asterisk (*) denotes non-specific band in nuclear fraction. **c** Quantification of PGRN expression in WT or TFEB-GFP HeLa lysates in b. (*n* = 3 independent experiments). **d** TFEB-GFP HeLa cells were treated with trehalose (100 mM) or Torin1 (250 nM) for 0, 2, or 24 h and imaged live with a fluorescent microscope to visualize TFEB-GFP localization. **e** TFEB-GFP HeLa cells were treated with vehicle, trehalose (100 mM), or Torin1 (250 nM) for 24 h and then fractionated into cytoplasmic/membrane and nuclear extracts and immunoblotted for total TFEB and PGRN. Asterisk (*) denotes non-specific band in nuclear fraction. **f** Quantification of nuclear TFEB (top) and cytoplasmic/membrane PGRN (bottom) from the immunoblots in e. (*n* = 3 independent experiments). **g** Immunoblots from whole-cell lysates of HAP1 WT and TFEB KO cells showing absence of TFEB expression. **h** *GRN* mRNA levels were quantified from HAP1 wild-type (WT) and TFEB knock-out (TFEB KO) cell lines after treatment with vehicle or trehalose (100 mM) for 18 h (*n* = 3 independent experiments run in duplicate). **i** HAP1 WT and TFEB KO cells were treated with vehicle or trehalose (100 mM) for 24 h and then fractionated into cytoplasmic/membrane and nuclear extracts. Immunoblots for PGRN (cytoplasmic/membrane) and TFEB (nuclear) are shown. **j** Quantification of PGRN (top) and TFEB (bottom) from the immunoblots in i (*n* = 3 independent experiments). In all graphs, the bars represent the mean ± SEM. For a, c; *differs from WT, *P* < 0.05, **P < 0.01, using unpaired two-tailed Student’s *t*-test. For **f**, **h**, **j**; *P < 0.05, **P < 0.01, ***P < 0.001, ****P < 0.0001 using one-way ANOVA followed by Tukey’s comparison *post-hoc* test

The transcription factor EB (TFEB) has been described as a master regulator of autophagy-lysosomal gene expression [26, 27] and implicated in regulation of *GRN* transcription [26, 28]. In addition, loss of PGRN has been linked to TFEB activation [29] and trehalose has been reported to activate TFEB in cultured cells [30, 31]. Therefore, we sought to explore whether TFEB plays a role in mediating the trehalose-induced upregulation of PGRN. First, we measured the levels of *GRN* mRNA and PGRN protein in cultured HeLa cells stably overexpressing TFEB-GFP [12] compared to wild-type (WT) HeLa cells. We found that the TFEB-GFP HeLa cells had a small, but significant increase in both *GRN* mRNA and PGRN protein compared to wild-type HeLa cells (Fig. 4a–c), indicating that TFEB overexpression modulates PGRN expression via direct or indirect mechanisms.

Under basal conditions, TFEB is normally phosphorylated by several kinases, including mTOR, which retains TFEB in the cytoplasm. Upon nutrient starvation or pharmacological inhibition of mTOR, TFEB is dephosphorylated and translocates to the nucleus where it activates transcription of its gene targets [12, 32, 33]. To test whether trehalose activates TFEB, we treated TFEB-GFP HeLa cells with Torin1 or trehalose and monitored TFEB-GFP localization using fluorescence microscopy. While Torin1 robustly increased TFEB nuclear localization by 2 h, trehalose treatment only resulted in a diffuse nuclear TFEB-GFP signal by 24 h of treatment (Fig. 4d). Fractionation of treated TFEB-GFP HeLa cells into cytoplasmic/membrane and nuclear fractions revealed that trehalose treatment increased nuclear TFEB levels slightly whereas Torin1 significantly increased nuclear TFEB levels (Fig. 4e–f). In contrast, trehalose treatment robustly increased PGRN levels, significantly more than Torin1 treatment (Fig. 4e–f), indicating that the amount of TFEB in the nucleus does not correlate with PGRN expression.

Finally, to directly address the role of endogenous TFEB on trehalose-mediated PGRN upregulation we utilized a human haploid cell line (HAP1) deficient in TFEB (TFEB KO) that was generated using CRISPR/Cas9. First, we confirmed by immunoblot that TFEB was not expressed in HAP1 TFEB KO cells (Fig. 4g). We then treated HAP1 wild-type (WT) or TFEB KO cells with vehicle or trehalose and measured *GRN* mRNA and PGRN protein levels. TFEB KO cells showed no significant decrease in *GRN* mRNA or PGRN protein levels compared to WT cells (Fig. 4h, j). However, trehalose treatment significantly increased *GRN* mRNA levels in both cell lines to similar levels (Fig. 4h). Accordingly, we found that trehalose was able to robustly increase PGRN protein expression in TFEB KO cells to the same level as in WT cells (Fig. 4i–j). Taken together, these results demonstrate that trehalose is a relatively weak activator of TFEB and that activation of TFEB is not responsible for the trehalose-induced upregulation of *GRN*/PGRN expression in cultured cells.

### Trehalose increases PGRN expression in *GRN* haploinsufficient patient-derived cells

**Fig. 5:**
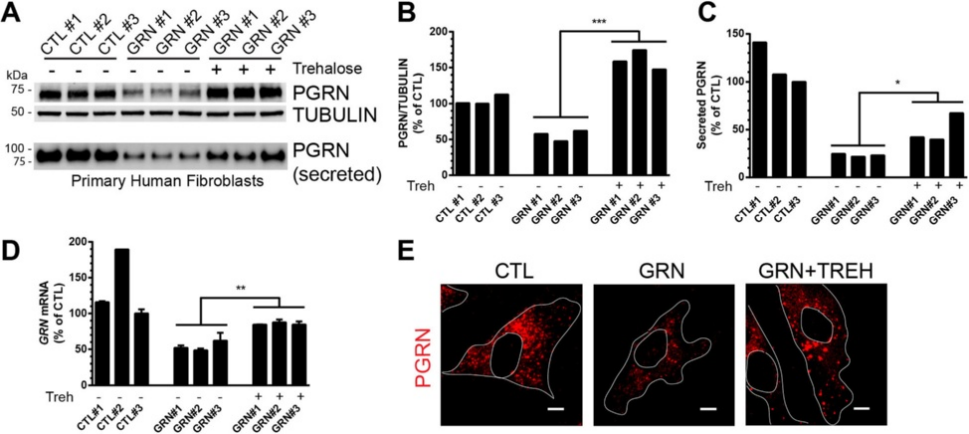
Trehalose rescues PGRN deficiency in patient-derived primary fibroblasts. **a** Top, Immunoblot of cell lysates from primary human fibroblast (3 controls; CTL, and 3 progranulin mutation; GRN) cell lines treated with vehicle or trehalose (100 mM) for 24 h. Bottom, Immunoblot of conditioned serum-free media from primary human fibroblast cell lines treated with vehicle or trehalose (100 mM) for 20 h. **b** Quantification of intracellular PGRN from immunoblots in a (top). **c** Quantification of secreted PGRN from immunoblots in a (bottom). **d** *GRN* mRNA levels were quantified from control cell lines (CTL) or progranulin mutation cell lines treated with vehicle or trehalose (100 mM) for 18 h (each individual line run in duplicate on qPCR). **e** GRN primary fibroblasts treated with trehalose (100 mM, 20 h) showed increased cytoplasmic staining of PGRN (red) similar to CTL fibroblasts. Scale bar, 10 μm. Nuclei (circles) and cell bodies are outlined in white. In all graphs, the data are normalized to the lowest CTL value for comparison. For **b** and **c**, bars represent individual densitometric measurements from the immunoblots in a and for d, bars represent the mean ± SEM of two qPCR replicate values. *Differs from untreated GRN, *P* < 0.05, **P < 0.01, ***P < 0.001, using unpaired two-tailed Student’s *t*-test

We then asked if trehalose could rescue PGRN deficiency in an in vitro *GRN* haploinsufficient model. Human primary fibroblast cell lines were generated from 3 control (CTL) or 3 progranulin mutation (GRN) carriers with the following *GRN* mutations: c.1477C > T (R493X) (designated GRN #1, GRN #2) and c.592_593delAG (R198GfsX19) (designated GRN #3). Intracellular PGRN protein was reduced ~50 % in the GRN lines compared to CTL lines (Fig. 5a–b). However, secreted PGRN levels were reduced by nearly 80 % in GRN lines compared to CTL lines (Fig. 5a, c), which is comparable to circulating PGRN levels in human patients with *GRN* mutations [34–36] and may reflect a defect in exosome secretion [37]. Treatment of GRN fibroblasts with trehalose significantly increased intracellular and secreted PGRN compared to vehicle treated GRN fibroblasts (Fig. 5a–c). Additionally, *GRN* mRNA from GRN fibroblasts, which was reduced by ~55 % compared to CTL fibroblasts, was significantly increased to near CTL levels after trehalose treatment (Fig. 5d). Immunocytochemistry of primary human fibroblasts revealed a robust increase in PGRN staining in GRN cells treated with trehalose, similar to vehicle treated CTL cells (Fig. 4e).

**Fig. 6:**
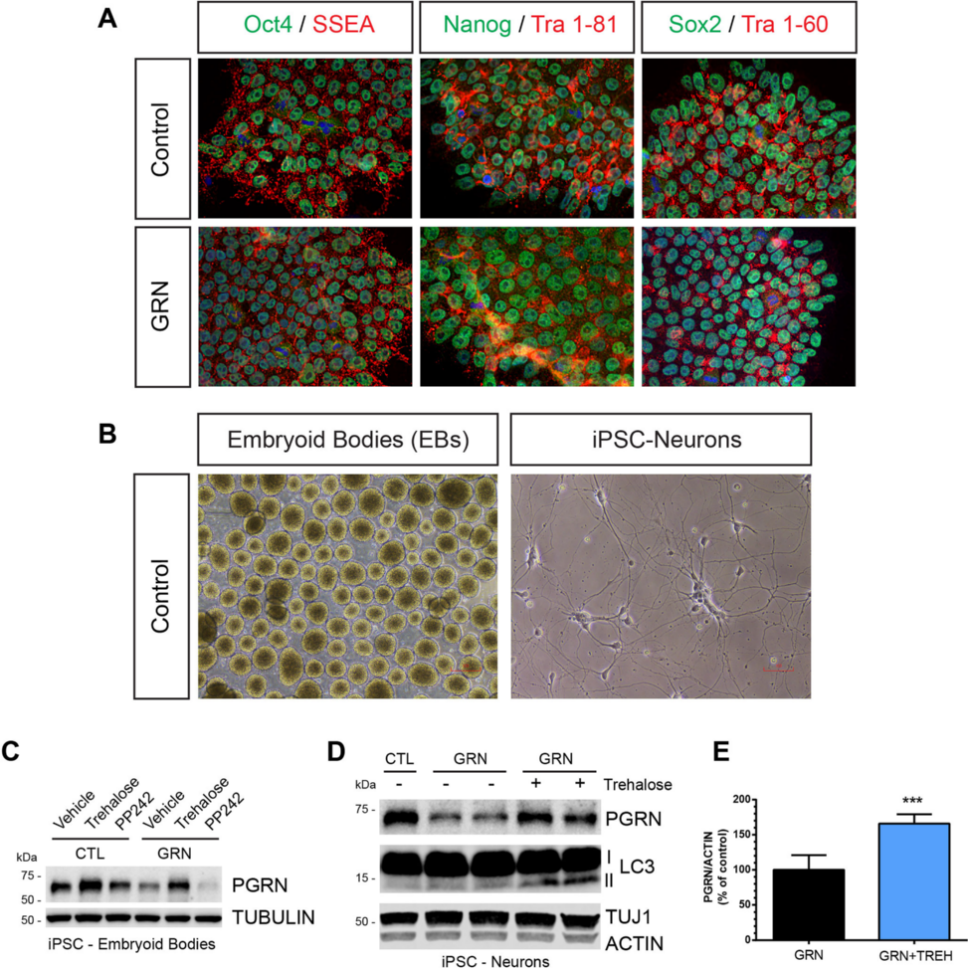
Trehalose rescues PGRN deficiency in induced pluripotent stem cell (iPSC) embryoid bodies (EBs) and iPSC-derived neurons. **a** Immunofluorescence verifies that iPSC colonies generated from control and *GRN*-mutation patients express the following standard pluripotent stem-cell markers: pluripotent transcription factors Oct4, Nanog, and Sox2 (green) and surface markers SSEA4, TRA 1–81, and TRA 1–60 (red). **b** Representative phase images of embryoid body (EB) formation during differentiation and highly pure iPSC-derived neurons approximately 1-week post plating. **c** Immunoblot of embryoid body cell lysates generated from one CTL and one GRN iPSC lines after treatment with vehicle, trehalose (100 mM), or PP242 (1 μM) for 24 h. **d** Representative Immunoblot of cell lysates from GRN iPSC-derived neurons treated with trehalose (100 mM) for 24 h. Two independent replicates from the GRN iPSC-neurons are shown. An untreated control (CTL) patient iPSC-neuron lysate was included to show relative differences in PGRN expression levels. **e** Quantification of PGRN expression from vehicle and trehalose treated GRN iPSC-neurons (*n* = 5 independent experiments for each). In all graphs, the bars represent the mean ± SEM. ***Differs from control, *P* < 0.001 using unpaired two-tailed Student’s *t*-test

Next, we reprogrammed one CTL and one GRN (GRN #3) fibroblast line to generate induced pluripotent stem cells (iPSC) and derived neurons from each line using an embryoid body-based differentiation protocol (Fig. 6a–b). Treatment of the GRN embryoid bodies (EBs) with trehalose restored PGRN to CTL levels, whereas PP242 failed to increase PGRN (Fig. 6c), similar to what we observed in neuronal-like cells. In GRN iPSC-neurons, PGRN expression was detected in the cell body and processes (Additional file 1: Figure S4a) and expression was reduced compared to the CTL line (Fig. 6d). Treatment of GRN iPSC-neurons with trehalose for 24 h lead to a significant increase in PGRN (Fig. 6d–e) and LC3-II (Fig. 6d and Additional file 1: Figure S4b), indicating autophagosome induction. Overall, these data show that trehalose can rescue PGRN deficiency in cell-based models of *GRN* haploinsufficiency, including iPSCs and iPSC-derived neurons.

### Trehalose increases PGRN expression in vivo in a mouse model of *Grn* haploinsufficiency

**Fig. 7:**
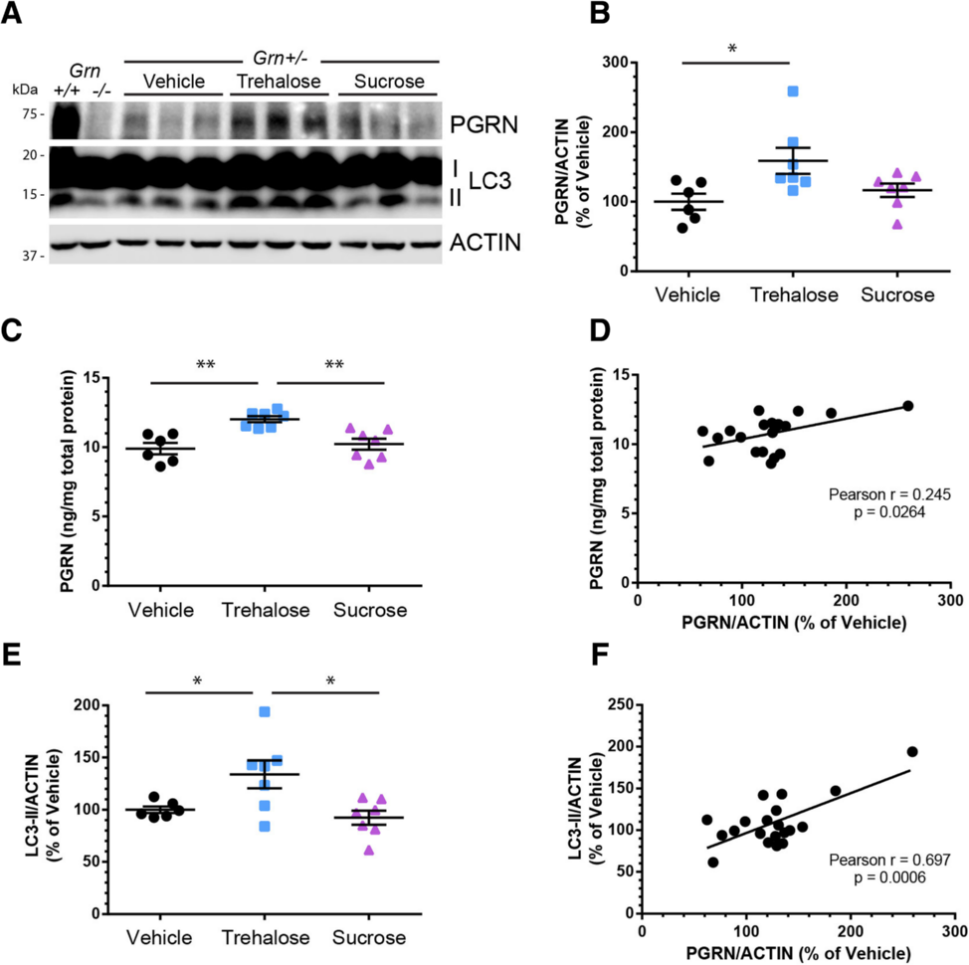
Oral trehalose administration increases PGRN and LC3-II in *Grn+/−* mouse brain. **a** Immunoblot of PGRN and LC3-II from brain lysates of treated *Grn+/−* animals. *Grn+/+* and *Grn−/−* brain lysates included as controls for PGRN immunoreactivity. **b** PGRN was significantly increased in trehalose treated mice compared to vehicle (*P < 0.05) or sucrose (*P* = 0.1) treated mice by immunoblot. **c** Brain PGRN was significantly increased in trehalose treated mice compared to vehicle (**P < 0.01) or sucrose (**P < 0.01) treated mice by ELISA. **d** Immunoblot and ELISA PGRN levels were positively and significantly correlated. **e** LC3-II was significantly increased in trehalose treated mice compared to vehicle (*P = 0.05) or sucrose (*P < 0.05) treated mice by immunoblot. **f** PGRN and LC3-II were positively and significantly correlated across all animals by immunoblot. In **b**, **c**, and **e**, each measurement per animal is plotted with the mean ± SEM shown for each group. Statistical differences were calculated by one-way ANOVA followed by Tukey’s comparison *post hoc* test

To extend our observations to an in vivo model, we treated three-month old *Grn+/−* mice with 2 % trehalose water (Trehalose, *n* = 7; 3 M/4 F) *ad libitum* for 65 days and then analyzed changes in PGRN levels in the brain and plasma. As controls, additional mice were treated with normal drinking water (Vehicle, *n* = 6; 3 M/3 F) or 2 % sucrose water (Sucrose, *n* = 7; 3 M/4 F). Similar treatment paradigms in mouse models have shown that trehalose can enhance autophagy [24, 38] and reduce pathologies [24, 39, 40], without adverse effects [41, 42]. In our study, mice treated with trehalose or sucrose consumed slightly more water than vehicle mice (Additional file 1: Figure S5a), although changes in body weight were not significant (Additional file 1: Figure S5b–c). Strikingly, PGRN levels in brain tissue were significantly increased in only the trehalose treated mice by both immunoblot (Fig. 7a–b) and ELISA (Fig. 7c), with both measures positively correlating (Fig. 7d). We also measured PGRN in plasma by ELISA but found no significant differences across groups (Additional file 1: Figure S5d). This discordance could be due to the fact that circulating plasma PGRN levels do not reflect brain/CSF PGRN levels [43, 44]. Finally, LC3-II was significantly increased in brain tissue of the trehalose treated mice (Fig. 7a, e), which was positively correlated with PGRN by immunoblot (Fig. 7f). Taken together, our results demonstrate that oral administration of trehalose increases brain PGRN levels and autophagic markers in vivo.

## Discussion

Reduced PGRN in the brain is linked to neurodegeneration in FTLD, AD, PD, and NCL. Therefore, increasing PGRN expression may be a viable therapeutic strategy to prevent or treat multiple neurodegenerative diseases. In support of this, expression of PGRN cDNA in an iPSC model of FTD-GRN rescued cortical neuron generation [45]. Further, viral mediated PGRN overexpression in the brain ameliorated neurodegeneration in a Parkinson’s disease model [46, 47] and reduced Aβ deposition and toxicity in an AD mouse model [48]. Despite these promising data, translating the delivery of PGRN into the brains of human patients is challenging. Alternatively, the discovery of small molecules that modulate PGRN expression would have tremendous therapeutic potential and also shed light on how PGRN expression is regulated.

Currently, there are only two published reports of small molecules that increase PGRN. First, alkalizing agents and inhibitors of the vacuolar ATPase (v-ATPase), such as chloroquine and bafilomycin A1 respectively, strongly enhance intracellular PGRN and secretion through an undefined post-transcriptional mechanism [25]. However, these molecules inhibit the autophagy-lysosome pathway [49, 50], which may exacerbate the disease process of FTLD-GRN. Second, select histone deacetylase inhibitors, in particular SAHA, increase PGRN through increased transcription [13]. Interestingly, SAHA also induces autophagy, possibly through inhibiting mTOR [51]. However, the molecular target(s) of SAHA are unclear and long-term administration may be toxic. Most critically, neither study evaluated the ability of the drugs to affect PGRN expression in neuronal or in vivo models of *GRN* haploinsufficiency. Because BafA1/chloroquine and SAHA both modulate the autophagy-lysosome pathway, we reasoned that other novel compounds that target this pathway may be used therapeutically to increase PGRN.

In this study, we generated a human *GRN* reporter construct to measure changes in PGRN expression in cells after treatment with a commercially available library of known autophagy-lysosome pathway modulators. We report that several novel compounds increased PGRN expression in various cell types, including several common mTOR inhibitors (rapamycin, PP242, Torin1) and the mTOR-independent activator of autophagy, trehalose. Torin1 and trehalose increased human *GRN* transcription reporter activity to levels comparable to SAHA (Fig. 1b). It should be noted that the reporter screen we employed only identified compounds that modulate *GRN* at the transcriptional level, which we confirmed by including bafilomycin A1 and chloroquine as negative controls. Therefore, additional compounds in this library may modulate PGRN expression via post-transcriptional mechanisms.

Our findings suggest that PGRN expression is upregulated in response to autophagy-lysosome pathway modulation, but is not dependent on mTOR inhibition, and is in line with growing evidence that PGRN plays a critical, yet undefined role in the cellular lysosomal pathway [10, 17, 18, 29, 52]. While mTOR inhibitors increased PGRN expression in some cell lines (HEK293T, H4, HeLa), they failed to raise PGRN in more neuronal cell types under the same treatment conditions, including neuroblastoma cells and primary mouse neurons, as well as human iPSC embryoid bodies. Previously, PGRN has been linked to modulating mTOR signaling pathways [29, 53, 54] indicating a possible feedback loop; however, further work is required to determine exactly how PGRN and the mTOR signaling pathway may regulate each other in various cell types. Despite the therapeutic promise of mTOR inhibitors for the treatment of aging and neurodegeneration, long term inhibition of mTOR is known to have detrimental side effects [55, 56]. Therefore, molecules that activate autophagy independent of mTOR inhibition may hold more therapeutic value for diseases that will require long-term dosing. Alternatively, trehalose and mTOR inhibitors may be used synergistically to treat neurodegeneration [20].

We focused on the compound trehalose for further study because it increased PGRN independent of mTOR inhibition in all cell lines tested, including patient fibroblasts and iPSC-derived neurons. Trehalose is a natural disaccharide found in bacteria, yeast, insects, fungi, and plants where it plays a critical role in stress and drought resistance; but it is not produced in vertebrates (reviewed in [42]). It is comprised of two α, α-1, 1-glycosidic-linked D-glucose molecules and is 45 % as sweet as sucrose. Trehalose is not easily hydrolyzed and does not react with free amino groups in non-enzymatic glycation reactions. It is used in some food products as a stabilizer and has been designated Generally Regarded as Safe (GRAS) by the Food and Drug Administration (FDA). As a neuroprotectant, trehalose increases autophagic flux in neurons both in vitro [57] and in vivo [58] through an undefined mechanism and we report here a similar effect in primary neurons from mouse and GRN iPSC-derived neurons. Trehalose also reduces accumulation of commonly misfolded proteins, in vitro and in vivo, that cause neurodegeneration including Aβ [48, 59], tau [38, 57, 60, 61], polyglutamine aggregates [39], mutant huntingtin [20, 62, 63], mutant SOD1 [23, 64], α-synuclein [20, 65–67], prion protein [68, 69], and TDP-43 [70] via its molecular chaperone and autophagy-activating properties. It is also worth noting that trehalose clears lipofuscin, a lysosomal storage material found in FTLD-GRN and AD patients, via activation of autophagy [71]. Finally, trehalose also acts as an anti-oxidant and anti-inflammatory molecule in several in vitro and in vivo models [72–74]. As such, trehalose may provide therapeutic benefit for the treatment of degenerative diseases via multiple mechanisms. Trehalose is currently being tested in clinical trials for reversal of arterial aging (ClinicalTrials.gov Identifier: NCT01575288) and for treatment of Oculopharyngeal Muscular Dystrophy (ClinicalTrials.gov Identifier: NCT02015481) and Spinocerebellar Ataxia 3 (ClinicalTrials.gov Identifier: NCT02147886).

Our results indicate that trehalose increases PGRN protein levels, at least in part, by increasing *GRN* transcription, similar to SAHA [13]. However, additional mechanisms such as enhanced protein stability cannot be ruled out. It is unknown what transcription factor(s) may be involved in the trehalose-mediated upregulation of *GRN* expression, although we have shown that the transcription factor EB (TFEB) is not necessary for its effect. TFEB is strongly regulated by mTOR phosphorylation, so perhaps it is not surprising that trehalose, which does not inhibit mTOR, is not a robust activator of TFEB. Future work will focus on identifying additional transcription factors activated by trehalose which may provide further mechanistic insights into *GRN* transcriptional regulation and additional therapeutic targets.

This is the first report demonstrating the ability of a small molecule to enhance PGRN expression in patient-derived *GRN* deficient neurons, as well as in an in vivo mouse model of *Grn* haploinsufficiency. Remarkably, we saw a significant increase in endogenous PGRN and LC3-II expression in brain tissue of trehalose treated mice compared to vehicle or sucrose treated mice. The sucrose treated mice served as an important control as the lack of effect on PGRN in these mice indicates that disaccharide hydrolysis into glucose monomers was not responsible for the increased PGRN expression detected. Due to the fact that we used young (3 month old) *Grn+/−* mice which do not have any known pathological or behavioral abnormalities [75], we were not able to assess the effects of trehalose on these parameters. It would be interesting to test whether trehalose can prevent or reverse neuropathology or behavioral deficits in aged *Grn−/−* mice independent of modulating PGRN expression and is a focus of future studies. Currently, relatively high concentrations of trehalose are needed to stimulate autophagy in vitro due to its slow penetrance of cell membranes via fluid-phase endocytosis [20] and we found that similar trehalose concentrations were needed to induce PGRN expression. Similarly, it is unclear if trehalose crosses the blood–brain-barrier to reach the brain, although it has previously been reported to do so [39]. Identification or synthesis of novel trehalose derivatives that have increased metabolic stability [76], increased cell penetrance [77] or alternative modes of delivery such as intravenous (IV) infusion may result in lower doses and enhanced bioactivity needed to achieve PGRN upregulation and will be explored in future studies.

## Conclusions

In this study, we report the novel finding that trehalose induces PGRN expression in in vitro and in vivo models of PGRN deficiency. Trehalose has pleiotropic properties that make it an attractive neuroprotectant. Moreover, trehalose is FDA-approved and is currently being tested in several clinical trials as an autophagy modulator. Based on our current findings, we conclude that trehalose should be explored as a first-generation therapeutic treatment for frontotemporal dementia with *GRN* mutations as well as other neurodegenerative diseases such as AD and PD, where reduced PGRN may be a risk factor.

## Abbreviations

PGRN, progranulin; *GRN*, Granulin gene; FTLD, frontotemporal lobar degeneration; FTD, frontotemporal dementia; AD, Alzheimer’s disease; PD, Parkinson’s disease; iPSCs, Induced Pluripotent Stem Cells; NCL, neuronal ceroid lipofuscinosis; TDP-43, TAR DNA-binding protein 43; mTOR, mechanistic target of rapamycin; TFEB, transcription factor EB; GFP, Green fluorescent protein

## Additional file

Additional file 1: Table S1. List of top compounds identified from the autophagy-lysosome library that increase *GRN* reporter activity. *, S.D. = standard deviation; **, Z-score = (compound normalized activity – average normalized activity)/population S.D. **Figure S1.** Validation of mTOR inhibitors on PGRN expression in cultured cells. a Dose–response of mTOR inhibitors in H4 neuroglioma cells. Cells were treated for 24 h and whole cell lysates were analyzed for PGRN expression as well as autophagy induction (p62 and LC3-II). P-S6 and P-4EBP1 were used to monitor mTOR inhibition. Results are representative of two independent experiments. **Figure S2.** Trehalose treatment increases autophagic flux without affecting lysosomal acidification or cell viability. **a** Immunoblot of cell lysates from H4 cells showing increased autophagic flux with trehalose treatment. **b** Quantification of autophagic flux data in a, (*n* = 3 independent experiments). ***P* < 0.01, ****P* < 0.001, **** *P* < 0.0001 using one-way ANOVA followed by Tukey’s comparison *post-hoc* test. **c** LysoTracker red staining of live H4 cells after 24-h treatment with vehicle (DMSO), bafilomycin A1 (50 nM), or trehalose (50 mM and 100 mM). Scale bar, 100 μM. Images are representative of two independent experiments. **d** Dose- and time-dependent cell viability after trehalose treatment in H4 cells using trypan-blue exclusion assay, (*n* = 3 independent experiments measured in duplicate). Statistical differences were calculated by one-way ANOVA followed by Dunnet’s comparison *post-hoc* test. No significant differences were found for individual treatment groups compared to untreated control. In all graphs, the bars represent the mean ± SEM. **Figure S3.** Trehalose treatment increases PGRN expression in neuroblastoma cell lines in a dose- and time-dependent manner. **a** Immunoblot of cell lysates from human SH-SY5Y neuroblastoma cells treated with increasing concentrations of trehalose for 24 h. **b** Immunoblot of cell lysates from human SH-SY5Y cells treated with 100 mM trehalose for increasing times. **c** Imunoblot of cell lysates from murine N2a neuroblastoma cells treated with increasing concentrations of trehalose for 24 h. **d** *GRN* mRNA levels in SH-SY5Y cells after treatment with 100 mM trehalose for 18 h. ***P* < 0.01 using unpaired two-tailed Student’s *t*-test. Results are representative of two independent experiments. In all graphs, the bars represent the mean ± SEM. **Figure S4.** Characterization of iPSC-derived neurons from primary human fibroblast cultures. **a** iPSC-derived neurons from a GRN patient express PGRN (green) in cell bodies (arrow) and processes (arrowheads) and neuronal specific marker TUJ1 (magenta). Nuclei (blue) are stained with DAPI. Scale bar, 10 μm. **b** iPSC-derived neurons from a GRN patient treated with trehalose (100 mM) for 24 h show intense LC3-labeled puncta (arrows), indicating autophagosome formation, compared to vehicle treated GRN neurons. MAP2 (magenta) was used as a neuronal marker. Nuclei (blue) were labeled with DAPI. Scale bar, 10 μm. **Figure S5.** Oral trehalose treatment does not affect total water consumption, weight change, or plasma PGRN levels in *Grn*+/− mice. **a** Average daily water consumption in milliliters (ml) for mice in each treatment group. *****P* < 0.0001. **b** Body weight in grams (g) over time for each treatment group. **c** Quantification of change in body weight (g) for each treatment group over the duration of the study. **d** Plasma PGRN levels as determined by Adipogen ELISA. In all graphs, the bars represent the mean ± SEM. Statistical differences of trehalose or sucrose groups relative to vehicle group were calculated by one-way ANOVA followed by Tukey’s comparison *post-hoc* test. (DOCX 571 kb)
