# Supplementary material for: Trehalose upregulates progranulin expression in human and mouse models of GRN haploinsufficiency: a novel therapeutic lead to treat frontotemporal dementia
Source: Mol Neurodegener. 2016 Jun 24;11:46. doi: 10.1186/s13024-016-0114-3 (PMC4919863; doi:10.1186/s13024-016-0114-3)
Supplement: Additional file 1: Table S1. — List of top compounds identified from the autophagy-lysosome library that increase GRN reporter activity. *, S.D. = standard deviation; **, Z-score = (compound normalized activity – average normalized activity)/population S.D. Figure S1. Validation of mTOR inhibitors on PGRN expression in cultured cells. a Dose–response of mTOR inhibitors in H4 neuroglioma cells. Cells were treated for 24 h and whole cell lysates were analyzed for PGRN expression as well as autophagy induction (p62 and LC3-II). P-S6 and P-4EBP1 were used to monitor mTOR inhibition. Results are representative of two independent experiments. Figure S2. Trehalose treatment increases autophagic flux without affecting lysosomal acidification or cell viability. a Immunoblot of cell lysates from H4 cells showing increased autophagic flux with trehalose treatment. b Quantification of autophagic flux data in a, (n = 3 independent experiments). **P < 0.01, ***P < 0.001, **** P < 0.0001 using one-way ANOVA followed by Tukey’s comparison post-hoc test. c LysoTracker red staining of live H4 cells after 24-h treatment with vehicle (DMSO), bafilomycin A1 (50 nM), or trehalose (50 mM and 100 mM). Scale bar, 100 μM. Images are representative of two independent experiments. d Dose- and time-dependent cell viability after trehalose treatment in H4 cells using trypan-blue exclusion assay, (n = 3 independent experiments measured in duplicate). Statistical differences were calculated by one-way ANOVA followed by Dunnet’s comparison post-hoc test. No significant differences were found for individual treatment groups compared to untreated control. In all graphs, the bars represent the mean ± SEM. Figure S3. Trehalose treatment increases PGRN expression in neuroblastoma cell lines in a dose- and time-dependent manner. a Immunoblot of cell lysates from human SH-SY5Y neuroblastoma cells treated with increasing concentrations of trehalose for 24 h. b Immunoblot of cell lysates from human SH-SY5Y cells treated with 100 mM treha [file 13024_2016_114_MOESM1_ESM.docx]

**Supplemental Methods**

**LysoTracker staining**

H4 cells were plated in 35 mm dishes (In vitro Scientific). Two days later, cells were treated with vehicle (ultrapure Milli-Q® water), bafilomycin A1 (50 nM), or trehalose (50 mM or 100 mM) in DMEM for 20 hours. After treatment, the media was removed and the cells were washed 3× with PBS. LysoTracker Red (Life Technologies) was dissolved in DMEM media to a final concentration of 50 nM and incubated with the cells for 15 min at 37°C. After incubation, the media was removed and the cells were washed 3× in PBS and fresh DMEM was added to the cells. LysoTracker staining was immediately imaged using an EVOS FL Cell Imaging System.

**Cell viability**

H4 cells were plated at approximately 5.0 × 10^4^ cells in a 12-well plate. Two days after plating, cells were treated with trehalose for the indicated times and concentrations in triplicate. After treatment, the cells were collected and transferred to a 1.5-mL microcentrifuge tube. A 1:1 aliquot of TC10 trypan blue dye (Bio-Rad) and cells were pipetted up and down 10 times and 10 µL of cells were applied to a TC10 counting slide (Bio-Rad) and counted on a TC10 automated Cell Counter (Bio-Rad). Counts were performed in duplicate and the % of viable cells (live/dead) was recorded.

**Autophagic flux assay**

H4 cells were plated at approximately 5.0 × 10^4^ cells in a 12-well plate. Two days after plating, cells were treated with trehalose or vehicle for 20 hours followed by co-treatment with or without bafilomycin A1 (100 nM) for an additional 4 hours to block autophagic flux. The concentration of BafA1 used was found to be saturating for LC3-II formation in H4 cells in previous experiments (data not shown).

**Supplemental Table**

**Table. S1** List of top compounds identified from the autophagy-lysosome library that increase *GRN* reporter activity. *, S.D. = standard deviation; **, Z-score = (compound normalized activity – average normalized activity)/population S.D.

| **Compound** | **Fold Change** | **S.D.*** | **Z-score**** | **Target or Pathway** | **Reference** |
| --- | --- | --- | --- | --- | --- |
| Torin 1 | 2.18 | 0.12 | 6.63 | mTOR | [1] |
| Trehalose | 2.00 | 0.03 | 5.34 | Unknown; mTOR-independent | [2] |
| SAHA | 1.80 | 0.62 | 3.95 | HDAC; mTOR | [3-5] |
| Imiquimod | 1.72 | 0.76 | 3.38 | TLR7 | [6] |
| STF-62247 | 1.57 | 0.40 | 2.31 | Golgi? | [7] |
| PI-103 | 1.50 | 0.03 | 1.84 | PI3K/mTOR | [8] |
| Capsaicin | 1.48 | 0.28 | 1.65 | TRPV1; Ca(2+); PI3K/AKT/mTOR | [9-11] |
| Curcumin | 1.46 | 0.01 | 1.54 | AKT/mTOR | [12, 13] |
| Deoxycholate·Na | 1.46 | 0.19 | 1.50 | Unknown | [14] |
| SB202190 | 1.44 | 0.04 | 1.39 | p38 MAPK; mTOR | [15] |
| Trifluoperazine·2HCl | 1.43 | 0.05 | 1.32 | Calmodulin; mTOR | [16, 17] |
| Rapamycin | 1.40 | 0.03 | 1.12 | mTORC1 | [18, 19] |

**Supplemental Figures**

**
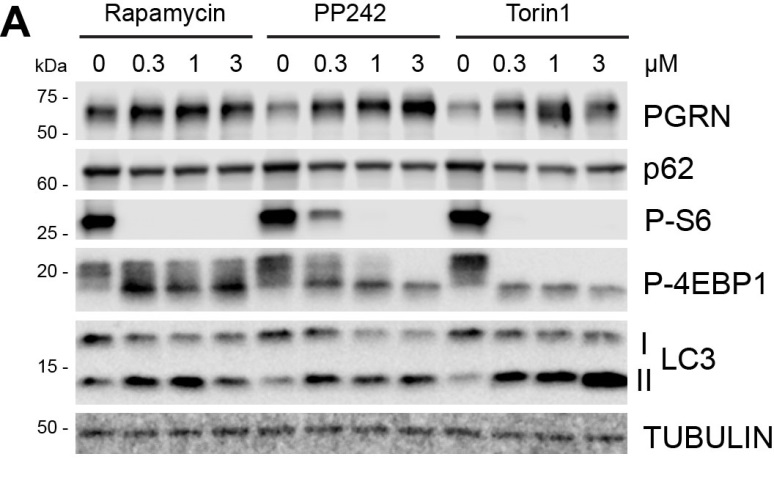
**

**Fig. S1** Validation of mTOR inhibitors on PGRN expression in cultured cells. **a** Dose-response of mTOR inhibitors in H4 neuroglioma cells. Cells were treated for 24 hours and whole cell lysates were analyzed for PGRN expression as well as autophagy induction (p62 and LC3-II). P-S6 and P-4EBP1 were used to monitor mTOR inhibition. Results are representative of two independent experiments.

**
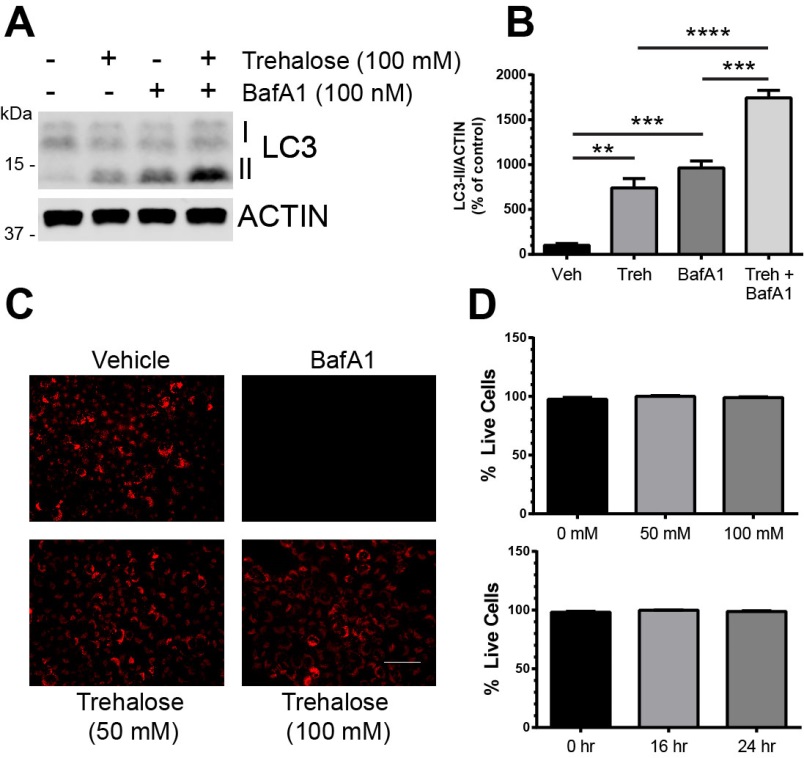
**

**Fig. S2** Trehalose treatment increases autophagic flux without affecting lysosomal acidification or cell viability. **a** Immunoblot of cell lysates from H4 cells showing increased autophagic flux with trehalose treatment. **b** Quantification of autophagic flux data in a, (*n* = 3 independent experiments). ***P* < 0.01, ****P* < 0.001, **** *P* < 0.0001 using one-way ANOVA followed by Tukey’s comparison *post-hoc* test. **c** LysoTracker red staining of live H4 cells after 24-hour treatment with vehicle (DMSO), bafilomycin A1 (50 nM), or trehalose (50 mM and 100 mM). Scale bar, 100 µM. Images are representative of two independent experiments. **d** Dose- and time-dependent cell viability after trehalose treatment in H4 cells using trypan-blue exclusion assay, (*n* = 3 independent experiments measured in duplicate). Statistical differences were calculated by one-way ANOVA followed by Dunnet’s comparison *post-hoc* test. No significant differences were found for individual treatment groups compared to untreated control. In all graphs, the bars represent the mean ± SEM.

**
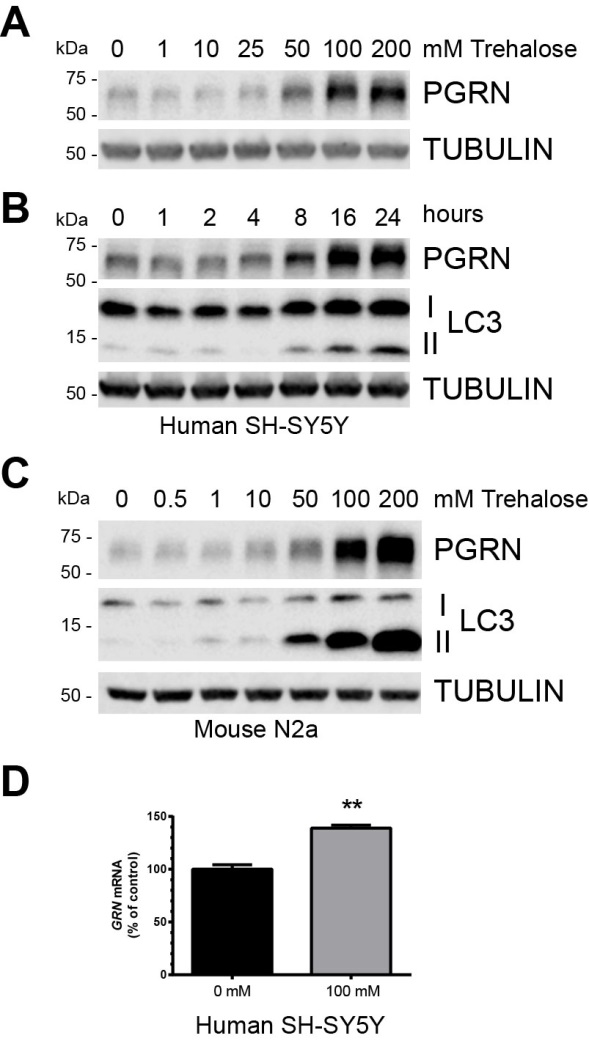
**

**Fig. S3** Trehalose treatment increases PGRN expression in neuroblastoma cell lines in a dose- and time-dependent manner. **a** Immunoblot of cell lysates from human SH-SY5Y neuroblastoma cells treated with increasing concentrations of trehalose for 24 hours. **b** Immunoblot of cell lysates from human SH-SY5Y cells treated with 100 mM trehalose for increasing times. **c** Imunoblot of cell lysates from murine N2a neuroblastoma cells treated with increasing concentrations of trehalose for 24 hours. **d** *GRN* mRNA levels in SH-SY5Y cells after treatment with 100 mM trehalose for 18 hours. ***P* < 0.01 using unpaired two-tailed Student’s t-test. Results are representative of two independent experiments. In all graphs, the bars represent the mean ± SEM.

**
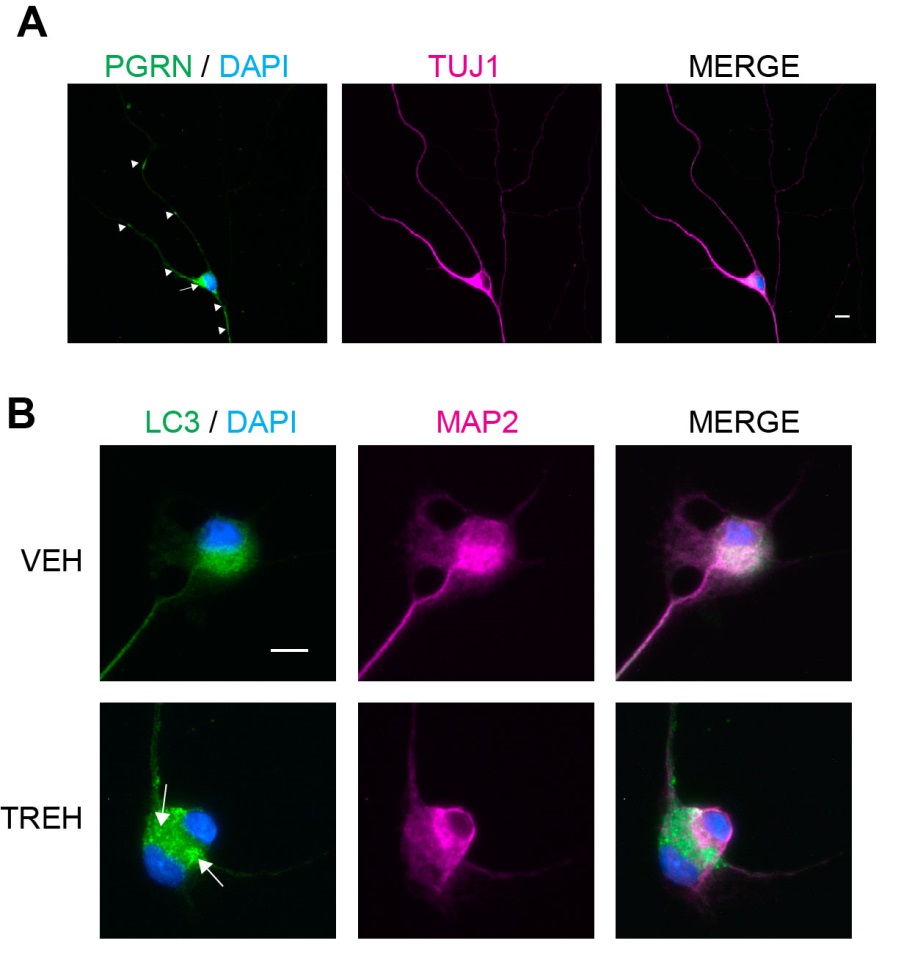
**

**Fig. S4** Characterization of iPSC-derived neurons from primary human fibroblast cultures. **a** iPSC-derived neurons from a GRN patient express PGRN (green) in cell bodies (arrow) and processes (arrowheads) and neuronal specific marker TUJ1 (magenta). Nuclei (blue) are stained with DAPI. Scale bar, 10 µm. **b** iPSC-derived neurons from a GRN patient treated with trehalose (100 mM) for 24 hours show intense LC3-labeled puncta (arrows), indicating autophagosome formation, compared to vehicle treated GRN neurons. MAP2 (magenta) was used as a neuronal marker. Nuclei (blue) were labeled with DAPI. Scale bar, 10 µm.

**
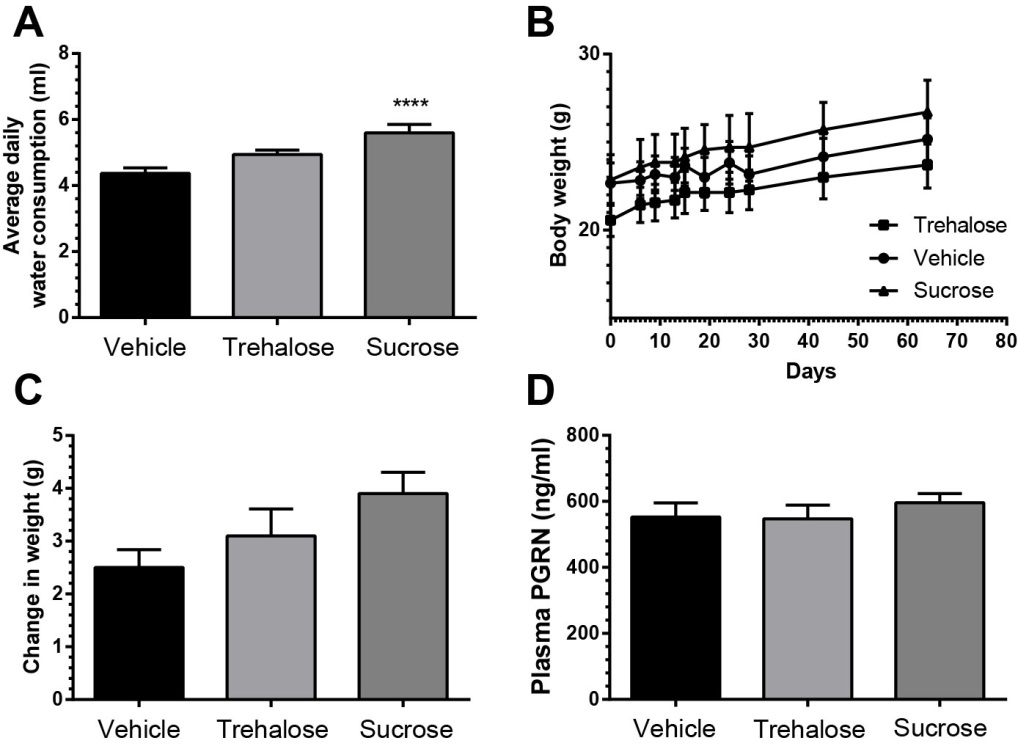
**

**Fig. S5** Oral trehalose treatment does not affect total water consumption, weight change, or plasma PGRN levels in *Grn*+/- mice. **a** Average daily water consumption in milliliters (ml) for mice in each treatment group. *****P* < 0.0001. **b** Body weight in grams (g) over time for each treatment group. **c** Quantification of change in body weight (g) for each treatment group over the duration of the study. **d** Plasma PGRN levels as determined by Adipogen ELISA. In all graphs, the bars represent the mean ± SEM. Statistical differences of trehalose or sucrose groups relative to vehicle group were calculated by one-way ANOVA followed by Tukey’s comparison *post-hoc* test.

**Supplemental References**

1. Thoreen CC, Kang SA, Chang JW, Liu Q, Zhang J, Gao Y et al. An ATP-competitive mammalian target of rapamycin inhibitor reveals rapamycin-resistant functions of mTORC1. The Journal of biological chemistry. 2009;284(12):8023-32. doi:10.1074/jbc.M900301200.

2. Sarkar S, Davies JE, Huang Z, Tunnacliffe A, Rubinsztein DC. Trehalose, a novel mTOR-independent autophagy enhancer, accelerates the clearance of mutant huntingtin and alpha-synuclein. The Journal of biological chemistry. 2007;282(8):5641-52. doi:10.1074/jbc.M609532200.

3. Kurundkar D, Srivastava RK, Chaudhary SC, Ballestas ME, Kopelovich L, Elmets CA et al. Vorinostat, an HDAC inhibitor attenuates epidermoid squamous cell carcinoma growth by dampening mTOR signaling pathway in a human xenograft murine model. Toxicology and applied pharmacology. 2013;266(2):233-44. doi:10.1016/j.taap.2012.11.002.

4. Chiao MT, Cheng WY, Yang YC, Shen CC, Ko JL. Suberoylanilide hydroxamic acid (SAHA) causes tumor growth slowdown and triggers autophagy in glioblastoma stem cells. Autophagy. 2013;9(10):1509-26. doi:10.4161/auto.25664.

5. Behera J, Jayprakash V, Sinha BN. Histone deacetylase inhibitors: a review on class-I specific inhibition. Mini reviews in medicinal chemistry. 2015;15(9):731-50.

6. Hemmi H, Kaisho T, Takeuchi O, Sato S, Sanjo H, Hoshino K et al. Small anti-viral compounds activate immune cells via the TLR7 MyD88-dependent signaling pathway. Nature immunology. 2002;3(2):196-200. doi:10.1038/ni758.

7. Turcotte S, Sutphin PD, Giaccia AJ. Targeted therapy for the loss of von Hippel-Lindau in renal cell carcinoma: a novel molecule that induces autophagic cell death. Autophagy. 2008;4(7):944-6.

8. Zou ZQ, Zhang XH, Wang F, Shen QJ, Xu J, Zhang LN et al. A novel dual PI3Kalpha/mTOR inhibitor PI-103 with high antitumor activity in non-small cell lung cancer cells. International journal of molecular medicine. 2009;24(1):97-101.

9. Caterina MJ, Schumacher MA, Tominaga M, Rosen TA, Levine JD, Julius D. The capsaicin receptor: a heat-activated ion channel in the pain pathway. Nature. 1997;389(6653):816-24. doi:10.1038/39807.

10. Hong ZF, Zhao WX, Yin ZY, Xie CR, Xu YP, Chi XQ et al. Capsaicin Enhances the Drug Sensitivity of Cholangiocarcinoma through the Inhibition of Chemotherapeutic-Induced Autophagy. PloS one. 2015;10(5):e0121538. doi:10.1371/journal.pone.0121538.

11. Chien CS, Ma KH, Lee HS, Liu PS, Li YH, Huang YS et al. Dual effect of capsaicin on cell death in human osteosarcoma G292 cells. European journal of pharmacology. 2013;718(1-3):350-60. doi:10.1016/j.ejphar.2013.08.011.

12. Zhao G, Han X, Zheng S, Li Z, Sha Y, Ni J et al. Curcumin induces autophagy, inhibits proliferation and invasion by downregulating AKT/mTOR signaling pathway in human melanoma cells. Oncology reports. 2015. doi:10.3892/or.2015.4413.

13. Shinojima N, Yokoyama T, Kondo Y, Kondo S. Roles of the Akt/mTOR/p70S6K and ERK1/2 signaling pathways in curcumin-induced autophagy. Autophagy. 2007;3(6):635-7.

14. Roesly HB, Khan MR, Chen HD, Hill KA, Narendran N, Watts GS et al. The decreased expression of Beclin-1 correlates with progression to esophageal adenocarcinoma: the role of deoxycholic acid. American journal of physiology Gastrointestinal and liver physiology. 2012;302(8):G864-72. doi:10.1152/ajpgi.00340.2011.

15. Menon MB, Kotlyarov A, Gaestel M. SB202190-induced cell type-specific vacuole formation and defective autophagy do not depend on p38 MAP kinase inhibition. PloS one. 2011;6(8):e23054. doi:10.1371/journal.pone.0023054.

16. Zhang L, Yu J, Pan H, Hu P, Hao Y, Cai W et al. Small molecule regulators of autophagy identified by an image-based high-throughput screen. Proceedings of the National Academy of Sciences of the United States of America. 2007;104(48):19023-8. doi:10.1073/pnas.0709695104.

17. Xu B, Chen S, Luo Y, Chen Z, Liu L, Zhou H et al. Calcium signaling is involved in cadmium-induced neuronal apoptosis via induction of reactive oxygen species and activation of MAPK/mTOR network. PloS one. 2011;6(4):e19052. doi:10.1371/journal.pone.0019052.

18. Brown EJ, Albers MW, Shin TB, Ichikawa K, Keith CT, Lane WS et al. A mammalian protein targeted by G1-arresting rapamycin-receptor complex. Nature. 1994;369(6483):756-8. doi:10.1038/369756a0.

19. Johnson SC, Rabinovitch PS, Kaeberlein M. mTOR is a key modulator of ageing and age-related disease. Nature. 2013;493(7432):338-45. doi:10.1038/nature11861.
